# Correlation between traumatic pelvic ring injuries and sexual dysfunctions: a multicentric retrospective study

**DOI:** 10.1007/s00264-023-05767-0

**Published:** 2023-03-17

**Authors:** Giuseppe Rovere, Amarildo Smakaj, Andrea Perna, Domenico De Mauro, Lorenzo Are, Luigi Meccariello, Andrea Fidanza, Rocco Erasmo, Francesco Falez, Giulio Maccauro, Francesco Liuzza

**Affiliations:** 1 Orthopaedic Institute, Fondazione Policlinico Universitario A. Gemelli IRCCS - Università Cattolica del Sacro Cuore, Rome, Italy; 2 Department of Orthopaedics and Traumatology, Fondazione Casa Sollievo della Sofferenza, San Giovanni Rotondo, Italy; 3 Department Orthopaedics and Traumatology, AORN San Pio, Benevento, Italy; 4 Department of Orthopaedics and Traumatology, Santo Spirito Hospital, Pescara, Italy; 5 Department of Orthopaedics and Traumatology, S. Filippo Neri Hospital, ASL Roma 1, Rome, Italy

**Keywords:** Pelvis, Fracture, Fracture fixation, Physiological sexual dysfunction, Lumbosacral plexus

## Abstract

**Purpose:**

Among the functional impairments associated with pelvic ring injuries (PRI), sexual dysfunction (SD) is a common clinical issue. The aim of this study is to investigate correlations between traumatic PRI, genitourinary, and sexual dysfunctions, for a proper multidisciplinary treatment.

**Methods:**

We performed an observational, multicentric study, from January 2020 to 2022. We conducted a follow-up after surgery at three, six, 12, and 24 months by measuring the Female Sexual Functioning Index (FSFI), the International Index of Erectile Function (IIEF), the Arizona Sexual Experience Scale (ASEX), the Majeed Score, and the SF-12. Descriptive statistics was conducted on *T*-test, Whelc’s test, and one-way ANOVA which were performed when appropriate.

**Results:**

A total of 76 patients (mean age 42.17 ± 15 years) were included in the study and allocated into three groups (A, B, and C). Tile A group revealed good sexual outcomes, similar to that of healthy patients. Tile B group demonstrated worsen SD than the previous group. In Tile C group, there was a longer average duration of the orthopaedic surgery when compared to group B. However, in terms of SDs, statistical significance could not be demonstrated between groups C and B.

**Conclusions:**

We observed a progressive spontaneous recovery of sexual function, corresponding to each PRI group. Moreover, men classified as B2 had milder SDs than B1 male patients.

## Introduction

Traumatic pelvic ring injuries (PRI) are an uncommon event in general population as they represent 3 to 8% of all fractures. As concerns complications, genitourinary injuries (GUI) are frequently associated with PRI due to the close anatomical relationship between the skeletal system, pelvic organs, and neurovascular structures, leading to a significantly decrease in the quality-of-life, especially in younger patients [1–3]. Among the functional impairments associated with pelvic ring fractures, sexual dysfunction (SD) is a common problem, occurring as a result of indirect trauma to the neurovascular structures contained in the pelvic cavity [3, 4]. Moreover, as PRI are more frequent in the young population as a consequence of road traffic accidents, SDs stand as severe long-term complications of pelvic ring fractures. However, SDs are often unaddressed even in young, sexually active patients, leading to depression and reduction in the quality of life (QoL) [2–5].

To date, there is no general consensus about the relationship between the type of pelvic fracture, genitourinary lesions, and the incidence of SDs [6, 7], as the data in the literature are fragmentary and inconclusive.

In many cases, the genitourinary lesion causing SDs is not recognizable, suggesting that the damage may affect a nerve structure of the pudendal plexus (S2-S4). These lesions are difficult to be identified, but they represent the neurovascular aetiology of SDs [8, 9].

The aim of this study is to investigate if there is any correlation between traumatic PRI and genitourinary sexual dysfunctions, in order to support early multidisciplinary treatments in high-risk patients. Our hypothesis is that sexual outcomes after PRI are correlated to the fracture patter which is a consequence of the mechanism of trauma.

## Material and methods

### Study settings and design

This is a retrospective longitudinal cohort study. The present investigation was performed on patients referred to our institutions after a PRI. All data collected were analysed from January 2020 to January 2022. All patients were recruited from multiple hospitals from the network of the Osteosynthesis and Trauma Care Association Italy. At hospital admission, all patients signed a written consent concerning demographic and clinical data for scientific purposes according to institutional protocols. The study respects national ethical standards and the Helsinki Convention and was approved by the Osteosynthesis and Trauma Care Association Italy (OTC Italy).

### Inclusion and exclusion criteria

Inclusion criteria were (I) the presence of osteo-ligamentous lesions of the pelvic rings A, B, and C according to tile fractures; (II) patients aged between 18 and 75 years; and (III) at least two year follow-up.

Exclusion criteria were (I) patients with previous urogenital or sexual disorders; (II) neurodegenerative, oncological, or haematological diseases; (III) previous pelvic ring orthopaedic surgery; and (IV) age under 18 or over 75. Patients were divided into groups according to the tile classification and its subgroups.

### Institutional database, data collection, and patients setting

Personal and demographical data were collected from all patients enrolled in the study. Hospital records were reviewed to determine the type of the trauma, the presence of acute associated injuries, and intraoperative and perioperative data. Local and systemic complications related to surgery were recorded.

All patients referred to the emergency departments (EDs) of the involved institutions had the following examinations as standard of care: antero-posterior view X-ray, inlet–outlet view X-ray, and computer tomography (CT) scan with two dimension (D) and 3D multiplanar reconstructions. The 3D reconstructions were useful to understand the number and position of bone fragments and the involvement of sacral foramina, and therefore to assess the possible degree of nerve involvement. The CT scan included the lumbar spine to identify possible vertebral fractures. Based on the above mentioned images, fractures were classified according to tile classification [10].

### Clinical and functional evaluation

Quality of life (QoL) and sexual dysfunction extent were assessed, at three, six, 12, and 24 months after surgery, using Female Sexual Functioning Index (FSFI) [11], International Index of Erectile Function (IIEF) [12], Arizona Sexual Experience Scale (ASEX) [13, 14], Majeed Score [15] and SF-12. These scores assess the presence and the extent of pain, work capacity, sexual function, independent movement, and psychological status [16].

### Statistical analysis

Data are presented as mean and standard deviation for continuous variables and frequency distribution (%) for categorical variables. Missing data were addressed by accepting them. Potential sources of bias were addressed by fidelity to the inclusion criteria protocol, avoidance of unintended interventions or co-interventions, obtaining complete data, and reporting of prespecified outcomes. The statistical analysis was performed using the Student’s *t* test for continuous variables and a chi-squared test for categorical variables. One-factor analysis of variance (ANOVA test) was used to compare subgroup functional outcomes (SF-12, Majeed, ASEX, FSFI, IIEF). Shapiro–Wilk test was used to test the normality of data. The significance was established for a value of *p* < 0.05. Dedicated GraphPad Prism (Version 9.4.1) statistical calculator program was employed.

## Results

A total of 76 patients (26 women and 50 men) with a mean age of 42.17 ± 15 years were enrolled in the study. None of the included patients was lost in the follow-up. Patients were allocated in fracture pattern subgroups according to the tile classification (Table 1). Traffic accidents were the main cause of pelvic fractures (68.4%). Surgical treatment was carried out with open reduction and internal fixation (ORIF) technique (32.9%), closed reduction and internal fixation (CRIF) (14.5), and mixed ORIF-CRIF (52.6%).

**Table 1:** Demographic, trauma mechanism, and surgical treatment data

	Tile A	Tile B	Tile C	Tot
Number of patients	3 (3.9%)	45 (59.2%)	28 (36.8)	76
Male/female ratio	3:0	2:1	17:11	25:13
Age	46.0 ± 4.0	42.5 ± 15.3	40.0 ± 16.4	41.7 ± 15.4
BMI	26.0 ± 3.4	26.9 ± 4.6	24.9 ± 5.1	26.1 ± 4.8
Injury mechanism				
Traffic accident	1	37	14	52 (68.4%)
Pedestrian hit by car	2	2	3	7 (9.2)
Fall from high	-	5	11	16 (21.0%)
Others	-	1	-	1 (1.3)
Surgical treatment				
ORIF	3	13	9	25 (32.9%)
CRIF	-	11	-	11 (14.5%)
ORIF + CRIF	-	21	19	40 (52.6%)
Operating time (min)	155.0 ± 35.4	135.8 ± 33.4	270.0 ± 143.0	173.8 ± 145.4
Time of hospitalization (days)	14.5 ± 2.1	24.4 ± 20.0	24.6 ± 34.6	29.4 ± 25.8

### Tile A

Two male patients were included in the Tile A group (Table 1). These patterns of fracture were relatively rare in our sample of patients, and good clinical outcomes were recorded. The mean hospitalization time was 14.5 ± 2.1 days. There were no sexual dysfunction recorded in this subgroup at 24-month follow-up, even after an initial impairment in the early postoperative months. These patients had complete motor recovery, as demonstrated by the results of the Majeed score (Table 3) and a high quality of life according to the SF-12 questionnaire (Table 3). Between the 12-month and 24-month follow-up, there was no significant clinical improvement (Table 3).

### Tile B

**Fig. 1:**
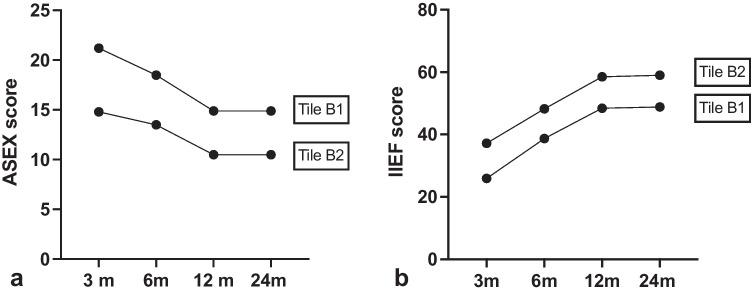
Comparison of ASEX M and IIEF values in male patients

**Fig. 2:**
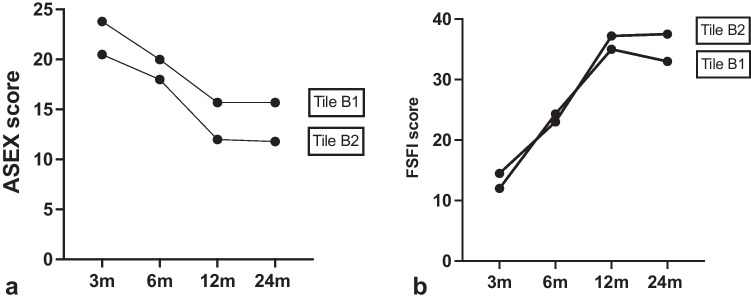
Comparison of ASEX F and FSFI values in female patients

The Tile B group includes the 59.2% of the enrolled patients (30 males and 15 females), of whom 23 had type B1 fracture, 18 type B2, and 4 type B3 fracture (Table 1). Questionnaires demonstrated a statistically significant improvement in clinical outcomes during the follow-up, as shown by ANOVA test (Tab. 2). The genitourinary deficits observed among the men included isolated erectile dysfunction in six patients, erectile dysfunction and urethral stenosis in five patients, and erectile dysfunction and urinary incontinence in two patients (Table 2). The most frequent disorders among females were isolated pelvic floor prolapse in three patients, pelvic floor prolapse and vulvodynia in four patients, and hypoaesthesia of the external genitalia in 1 patient (Table 2). The hospitalization of these patients was significantly longer than in the Tile A group (24.4 ± 20.0 days) as shown by Whelc’s test (*t* = 2743, df = 20.61, *p* = 0.01). Notably, at three month follow-up, male patients classified as B1 had worse mean index of sexual function according to IIEF and ASEX than B2 patients as shown in Fig. 1. This difference in women was less evident (Fig. 2), and FSFI values did not differ much between the two subgroups (33.0 ± 7.0 vs 35.7 ± 12.5).

**Table 2:** Genitourinary complications

	Tile B		Tile C	
	M	F	M	F
Erectile dysfunction	6	-	-	-
Erectile dysfunction and urethral stenosis	5	-	2	-
Erectile dysfunction and urinary incontinence	2	-	-	-
Pelvic floor prolapse	-	3	-	-
Pelvic floor prolapse and vulvodynia	-	4	-	-
Hypoesthesia of the external genitalia	-	2	-	-
Bladder injury	-	-	1	2

With regard to the B3 subgroup, it was not possible to draw significant results due to the small sample size. These patients revealed a poor motor recovery and QoL, as demonstrated by the results of the Majeed score (Table 3) and the SF-12 questionnaire (Table 3).

**Table 3:** Clinical outcomes. *Statistically significant (one-way ANOVA test)

	SF-12					Majeed score				
	3 m	6 m	12 m	24 m	*P* value	3 m	6 m	12 m	24 m	*P* value
Tile A	65.5 ± 0.7	78.0 ± 1.4	86.0 ± 1.4	95.0 ± 1.4	* < 0.01	56.5 ± 2.1	65.0 ± 2.8	85.5 ± 2.1	95.5 ± 0–7	0.06
Tile B	40.9 ± 10.7	62.1 ± 12.2	83.7 ± 7.3	83.6 ± 8.9	* < 0.01	28.3 ± 5.2	51.7 ± 7.7	70.9 ± 12.8	81.2 ± 7.4	* < 0.01
Tile B1	40.7 ± 11.8	62.1 ± 13.3	84.6 ± 7.6	84.4 ± 8.6	* < 0.01	29.2 ± 5.4	55.8 ± 7.5	79.5 ± 7.1	89.0 ± 5.0	* < 0.01
Tile B2	41.3 ± 8.1	62.1 ± 10.3	81.9 ± 6.6	82.1 ± 9.8	* < 0.01	30.7 ± 5.6	54.3 ± 6.0	79.0 ± 5.9	83.1 ± 3.0	* < 0.01
Tile B3	35.3 ± 6.4	61.3 ± 8.3	80.7 ± 7.0	78.0 ± 8.0	* < 0.01	25.0 ± 3.1	45.4 ± 6.0	55.4 ± 3.7	73.4 ± 4.5	* < 0.01
Tile C	36.6 ± 12.8	55.5 ± 16.3	70.6 ± 17.7	70.2 ± 18.4	* < 0.01	21.2 ± 2.4	32.0 ± 4.5	50.4 ± 6.2	75.6 ± 7.3	* < 0.01
Tile C1	37.8 ± 14.7	57.1 ± 19.6	73.0 ± 18.2	74.0 ± 18.5	* < 0.01	21.4 ± 2.7	33.4 ± 4.2	46.4 ± 4.8	70.6 ± 5.3	* < 0.01
Tile C2	36.8 ± 13.2	57.0 ± 13.8	72.8 ± 11.9	69.5 ± 10.8	* < 0.01	21.8 ± 2.5	32.3 ± 5.2	52.0 ± 7.0	77.7 ± 7.7	* < 0.01
Tile C3	32.5 ± 12.0	46.5 ± 17.7	58.0 ± 33.9	60.0 ± 36.8	* < 0.01	19.7 ± 1.5	29.0 ± 3.6	53.7 ± 4.5	79.7 ± 6.5	* < 0.01

At six months, an overall improvement in sexual outcomes was observed as shown by ASEX, IIEF, and FSFI results (Tables 1 and 2). At this time (6-month follow-up), the difference between B1 and B2 male patients was reduced, lasting better outcome in B2 patients (Fig. 1).

This difference was also noticed in women (Fig. 2): female B1 patient had a mean FSFI score of 24.3 ± 6.8 compared to a mean score of 23.0 ± 2.7 of the B2 subgroup. However, no statistical significance was found. Improvements in IIEF and FSFI scores were also observed, but this was not supported by statistical evidence.

At 12 months, the mean ASEX-M, IIEF, ASEX-F, and FSFI scores of B1 male patients were 14.9 ± 6.4, 48.4 ± 21.2, 15.7 ± 4.1, and 35.0 ± 7.6, respectively. Even one year after surgery, B2 patients had better ASEX, IIEF scores than B1 patients. This difference was observed also in the B2 female B2 group (Fig. 2).

At this time (12-month follow-up), the analysis of the Majeed and SF-12 questionnaires allowed to see progressive improvements (Table 3), without significant differences between the B1 and B2 subgroups.

At 24 months, no improvement in SF was observed compared to 12 months according to the ASEX questionnaire (Tables 3 and 4).

**Table 4:** Clinical outcomes female patients. *Statistically significant (one-way ANOVA test)

	FSFI					ASEX-F				
	3 m	6 m	12 m	24 m	*P* value	3 m	6 m	12 m	24 m	*P* value
Tile A	-	-	-	-	-	-	-	-	-	-
Tile B	13.3 ± 6.2	23.7 ± 5.0	36.1 ± 10.4	34.3 ± 9.8	* < 0.01	22.2 ± 6.5	19.0 ± 4.7	13.8 ± 3.7	13.8 ± 3.8	* < 0.01
Tile B1	12.0 ± 7.3	24.3 ± 6.8	35.0 ± 7.6	33.0 ± 7.0	* < 0.01	23.8 ± 6.9	20.0 ± 5.2	15.7 ± 4.1	15.7 ± 4.1	* < 0.01
Tile B2	14.5 ± 5.3	23.0 ± 2.7	37.2 ± 13.4	35.7 ± 12.5	* < 0.05	20.5 ± 6.2	18.0 ± 4.4	12.0 ± 2.2	11.8 ± 2.5	* < 0.05
Tile B3	-	-	-	-	-	-	-	-	-	-
Tile C	10.0 ± 10.6	21.5 ± 21.8	36.5 ± 23.8	37.5 ± 22.8	* < 0.05	26.5 ± 4.4	20.8 ± 7.5	15.8 ± 8.3	15.3 ± 8.5	* < 0.05
Tile C1	10	16	16	18	-	28	28	26	26	-
Tile C2	25	53	69	69	-	20	12	6	6	-
Tile C3	2.5 ± 0.7	8.5 ± 7.8	30.5 ± 12.0	31.5 ± 10.6	0.15	29.0 ± 1.4	21.5 ± 6.4	15.5 ± 2.1	14.5 ± 3.5	* < 0.05

Minimal improvements were observed in the IIEF and FESFI questionnaires. At this follow-up, the Majeed score was stable, showing no changes on the motor outcomes of patients, as well as no improvements in the psychomotor profile evaluated according to SF12.

At two year follow-up, the comparison between the B1 and B2 subgroups revealed that the SF in B1 patients were stably higher than the B2 subgroup (Tables 3 and 4).

### Tile C

Twenty-eight patients were included in study group C, 17 men and 11 women (Table 1). An outcome improvement was observed for all questionnaires until the last follow-up. Among urological injuries, bladder injury and erectile dysfunction and urethral stenosis were observed in three and two patients, respectively (Table 2). Urethral lesions occurred in male patients. The multi-organ involvement and the severity of the clinical picture in type C fractures justify the longer average time of surgery when compared with group B. The average length of hospital stay was also longer than other groups (24.6 ± 34.6). At three month follow-up (Tables 3 and 4), ASEX-M, ASEX-F, IIEF, and FSFI scores were 23.3 ± 5.2, 26.5 ± 4.4, 24.3 ± 11.9, and 10.0 ± 10.6, respectively, without any statistically significant differences between the C1 and C2 subgroups. However, the small sample size limited this type of subgroup investigation. The one-way ANOVA test of the IIEF and FSFI questionnaires showed no statistically significant differences compared to group B, whereas ASEX score in male patients was worse than the B group (23.3 ± 5.2 vs 19.2 ± 5.7) (Table 5).

**Table 5:** Clinical outcomes of male patients. *Statistically significant (one-way ANOVA test)

	IIEF					ASEX-M				
	3 m	6 m	12 m	24 m	*P* value	3 m	6 m	12 m	24 m	*P* value
Tile A	30.0 ± 22.6	63.0 ± 11.3	69.5 ± 6.4	70.0 ± 5.7	0.33	22.0 ± 4.2	10.0 ± 7.1	8.0 ± 4.2	7.5 ± 3.5	0.10
Tile B	29.7 ± 14.4	42.6 ± 20.4	52.4 ± 18.2	52.7 ± 18.2	* < 0.01	19.2 ± 5.7	16.6 ± 6.3	13.3 ± 5.7	13.3 ± 5.7	* < 0.01
Tile B1	25.9 ± 15.7	38.7 ± 23.8	48.4 ± 21.2	48.8 ± 21.2	* < 0.01	21.2 ± 5.5	18.5 ± 7.0	14.9 ± 6.4	14.9 ± 6.4	* < 0.01
Tile B2	37.2 ± 10.1	48.2 ± 11.9	58.5 ± 9.8	59.0 ± 10.6	* < 0.01	14.8 ± 4.4	13.5 ± 3.4	10.5 ± 2.3	10.5 ± 2.3	* < 0.01
Tile B3	35.0 ± 8.2	52.3 ± 4.9	61.0 ± 4.0	61.0 ± 4.0	* < 0.05	17.7 ± 3.8	13.0 ± 2.6	10.3 ± 2.5	10.3 ± 2.5	0.11
Tile C	24.3 ± 11.9	36.1 ± 18.2	40.9 ± 17.9	42.2 ± 18.5	* < 0.01	23.3 ± 5.2	17.8 ± 5.8	14.9 ± 4.7	14.6 ± 4.9	* < 0.01
Tile C1	27.0 ± 11.8	41.8 ± 15.6	48.2 ± 13.6	50.0 ± 15.0	* < 0.01	21.8 ± 5.3	17.2 ± 6.1	15.2 ± 4.8	14.2 ± 5.2	* < 0.01
Tile C2	24.2 ± 11.6	34.4 ± 19.5	38.0 ± 18.9	38.8 ± 18.0	* < 0.05	24.0 ± 5.1	18.2 ± 6.8	14.6 ± 5.7	14.6 ± 5.7	* < 0.01
Tile C3	8	10	12	12	-	29	18	15	15	-

At six months, the ASEX-M, ASEX-F, IIEF, and FSFI values were 17.8 ± 5.8, 20.8 ± 7.5, 36.1 ± 18.2, and 21.5 ± 21.8, respectively (Tables 3 and 4). These values differed little from the corresponding values in type B fractures; however, one-way ANOVA test revealed no statistical significance between the averages of the two groups in either males or females. The differences between C1 and C2 fracture type assessed by ASEX-M and ASEX-F were minimal (Tables 3 and 4), and not supported by statistical evidence.

At 12 months, the mean ASEX-M, ASEX-F, IIEF, and FSFI scores were 14.9 ± 4.7, 15.8 ± 8.3, 40.9 ± 17.9, and 36.5 ± 23.8 respectively. Statistical analysis showed a significant improvement for all three tests considered within one year from surgery. Statistical significance could not be demonstrated between groups C and B after one year follow-up for the ASEX and FSFI questionnaires. The differences observed by the IIEF were higher (52.4 ± 18.2 vs. 40.9 ± 17.9) although not within the 95% confidence interval (*p* = 0.08).

At 24 months, the ASEX-M, ASEX-F, IIEF, and FSFI values were 14.6 ± 4.9, 15.3 ± 8.5, 42.2 ± 18.5, and 37.5 ± 22.8, respectively, showing no significant changes compared to the previous follow-up.

The motor function also remained stable, showing that the regression of the motor deficit following the initial trauma stopped 12 months after the post-operative period (Table 3).

Even from the psychomotor point of view, a stability of the results according to the SF12 questionnaire was found (Table 3).

## Discussion

In this multicentric, longitudinal cohort study, we have identified that there is a correlation between long term SDs and the type of pelvic ring injuries by assessing ASEX, IIEF, and FSFI.

Sexual dysfunction (SD) is one of the long-term complications of pelvic fractures caused by high-energy trauma, yet it is often underestimated and underdiagnosed even in young, sexually active patients [14–17]. Furthermore, a correlation has been demonstrated between deficits in sexual function in pelvic trauma patients and reduced quality of life.

The pelvic fractures most commonly associated with sexual dysfunction are those involving the pubic branches, the pubic symphysis (B1-open book fracture), and injuries of the sacro-iliac joints [7, 18].

To date, there is no statistically significant correlation between tile classification and frequency or severity of SDs although our study showed that when comparing B1 and B2 patients, there are less severe deficits in the latter group. This could be since the B1 open-book fractures are externally rotating injuries in which there is an increase in the volume of the pelvic cavity. This volume increase could lead to tearing injuries of vessels and nerves. In B2 type fractures, the forces causing the trauma are on a latero-lateral axis resulting in an internal rotation displacement of the hemipelvis. In this condition, the volume of the pelvic cavity decreases and tearing injuries of nerves and vascular structures are less likely [19].

It seems clear that type A fractures show a lower frequency of SDs than type B and C fractures, in both males and females, because the continuity of the pelvic ring is not interrupted and they do not correlate with large pelvic organ injuries [4]. Mechanism of trauma, on which is based the tile classification, seems to be crucial in the incidence of SDs [20]. In fact, in our sample of patients, group A revealed a smaller number of associated injuries, and a significantly shorter hospitalization time when compared to groups B and C. This had a positive psychophysical impact on the patient, as evidenced by the excellent results of the SF-12 quality of life questionnaire, which could be protective against the onset of SD. In fact, Ozumba et. al 2004 [21] demonstrated how the various stress factors related to hospitalization can cause a reduction in sexual desire and libido.

In our study, injuries of the urethra and bladder affected up to 30% of patients. According to a recent review [7], after pelvic fractures, the incidence of genitourinary lesions associated with sexual dysfunction varies from 6.5 to 30%, which is in line with our analysis, which is 20%. Among the lesions observed, bladder lesions were the most frequent, followed by urethral and external genital lesions. Some authors estimate the incidence of genitourinary lesions between 8 and 21% after PRI, in a sample of men only [22]. In women, the incidence of associated genitourinary lesions is lower, about 6%, because the urethra is shorter and has not a tortuous course [23]. Those most affected organs in pelvic trauma are the bladder in both sexes and the urethra especially in the males, complicating up to 26% of pelvic fracture cases [5].

One of the most commonly investigated risk factors for the development of SDs following pelvic ring fractures is the presence and severity of associated urethral lesions [24]. In men, the most common site of urethral injury is the bulbous-membranous junction [25], where the urethra becomes fixed to mobile. However, the correlation between sexual disorders and genitourinary injuries is a much-debated topic, on which the literature is not clear. Traditionally, SDs may have both neurogenic or vascular aetiology. Erection is the most frequent sexual dysfunction and depends on a combination of psychosomatic stimuli.

In our sample of patients, women accounted for 29% only. In fact, female patients seem to be under represented. According to Vallier et al. [2], pain with sexual intercourse is common in women who have had a pelvic ring injury. Women with pelvic ring injury were more likely to report dyspareunia than other female patients with musculoskeletal trauma. However, as noted by Rovere et al. [7], there are fewer available data on sexual dysfunction in women after pelvic trauma. However, the integrity of the autonomic nerve pathway is also crucial in women as it controls the clitoral erection and the vaginal lubrication [26]. Neurogenic aetiology seems to be the main cause of SDs in patients suffering from pelvic ring injuries [8]; however, diagnostic tests that allow a neurophysiological investigation of pelvic nerve structures have poor application in clinical practice because of the complexity and operator dependence on the interpretation of results.

Among the vascular causes of SDs, it appears that a lack of blood supply is one of the most likely [18].

The main limitation of the study is the small sample size of enrolled patients which makes it impossible to detect statistical differences even if they are present. As this is a longitudinal retrospective cohort study, the main limitation is selection bias, mis-classification, or information bias. Study groups are not homogeneous in terms of demographics. Moreover, different types of surgical procedures were chosen in order to achieve a proper synthesis. Operations were not performed by a single surgeon.

## Conclusions

We observed a general and progressive spontaneous recovery of sexual functions throughout the follow-up period, corresponding to each PRI severity group. Within group B, according to clinical data and through the ASEX and IIEF questionnaires, men with B2 fractures had milder sexual dysfunction than males in the B1 group.

Larger cohort studies are desirable to answer opens questions on the issue and to suggest early appropriate multidisciplinary approaches of sexual disorders after pelvic ring fractures.

## Data Availability

Not applicable
